# Influence of oxygen on generation of reactive chemicals from nitrogen plasma jet

**DOI:** 10.1038/s41598-018-27473-3

**Published:** 2018-06-18

**Authors:** Han Sup Uhm, Se Hoon Ki, Ku Youn Baik, Eun Ha Choi

**Affiliations:** 1 New Industry Convergence Technology R&D Center, Ajou University, Suwon, 16499 Republic of Korea; 2 Electrical and Biological Physics, Kwangwoon University, Seoul, 01897 Republic of Korea

## Abstract

A nonthermal plasma jet is operated at atmospheric pressure inside a vacuum chamber filled with nitrogen gas. Various chemical compounds are fabricated from nitrogen and water molecules in plasma jet with varying oxygen content. Detailed theoretical investigation of these chemical compounds is carried out in terms of different oxygen ratio *ξ*. Experimental measurements are also carried out for comparison with theoretical results. Hydroxyl molecules are mostly generated at surface of water, and some of them can penetrate into water. The density of hydroxyl molecules has its maximum without oxygen, and decreases to zero as *ξ* increases to 0.25. The density of the ammonia of NH_3_ also deceases as *ξ* increases to 0.25. On the other hand, theory and experiment show that the density of the NO_3_ increases drastically as *ξ* increases to 0.25. The hydrogen peroxide density in plasma activated water deceases, reaches its minimum value at *ξ* = 0.05, and then increases again, as *ξ* increases from a small value to a large value. The *pH* value of the plasma activated water, which is slightly changed to alkali without oxygen, decreases as *ξ* increases.

## Introduction

Nonthermal plasma was proposed as a novel therapy for some incurable diseases^1–6^. Nonthermal plasma generates various kinds of reactive chemicals including reactive oxygen species (ROS) and reactive nitrogen species (RNS) in the liquid, and the consequent increase of intracellular ROS and RNS have been reported as main cause for various biological events. In recent studies, plasma activated water/media showed similar anticancer effects as direct nonthermal plasma treatment^7,8^. These reports support that those diseases may be cured by long lived ROS/RNS. H_2_O_2_ and ONOO^−^ have been suggested as main players in plasma treated liquids^8–10^. However, still it is unclear why any artificial combination of H_2_O_2_ and NO_2_^−^ cannot make full biological effects as plasma does. Therefore, the effects of different compositions of reactive species in the plasma need to be investigated.

Nitrogen plasma is known to produce OH radicals easily according to the water bombardment of excited nitrogen molecules in a metastable state^11^. Though OH radicals from the liquid surface are hard to directly affect biological systems, the OH radicals are important source to generate H_2_O_2_ or ONOO^−^. In addition, oxygen addition to the nitrogen gas causes dramatic changes in the chemistry of plasma treated water, resulting in differential biological responses^12–15^. Therefore, this article investigates influence of oxygen on generation of reactive species in nitrogen plasma.

In this study, detailed theoretical investigation of these chemical compounds is carried out in terms of different oxygen content (1~25%). The steady-state density of the H, OH, HO_2_, H_2_O_2_, NH, NH_2_, NH_3_, NO, HNO_2_, and HNO_3_ are calculated using major forty two chemical reactions (Table 1). Experimental measurements are also carried out in comparison with theoretical results. OH, NH_4_^+^, H_2_O_2_, NO_3_^−^, and pH are measured in deionized water treated with nonthermal plasma. Though our analysis does not show detail kinetics in a pico second range or detail spatial distributions in a micro meter range as other computational simulation studies, our steady-state solutions are well matched with experimental measurements. This is the first report showing the changes in the chemical species in water according to the O_2_ mole fraction in N_2_ plasma in both theoretical and experimental approaches. Densities of H, OH, H_2_O_2_, NH, NH_2_, and NH_3_ drastically reduced in the low level of O_2_ mole fraction, but densities of O and HNO_3_ increased according to O_2_ mole fraction. Their relations with the pH will be discussed.

**Table 1:** Chemical reactions used for theoretical approaches in this study.

Eq. Num.	Reaction	Rate coefficient	Ref.
1	N_2_ + e → N_2_(A_3_∑_u_^+^)	*α*_N2*_ = 6.4 × 10^−12^ cm^3^/s	
2	N_2_ + e → 2 N	*k*_*N*_ = 1 × 10^−12^ cm^3^/s	
3	O_2_ + e → 2 O	*k*_*O*_ = 1.5 × 10^−11^ cm^3^/s	
4	N_2_(A_3_∑_u_^+^) + H_2_O → OH + H + N_2_	*α*_*OH*_ = 5 × 10^−14^ cm^3^/s	^18^
5	N_2_(A_3_∑_u_^+^) + N_2_ → N_2_ + N_2_	*α*_*N2*_ = 3 × 10^−18^ cm^3^/molecule/s	^18^
6	N_2_(A_3_∑_u_^+^) + O_2_ → products	*α*_*O2*_ = 2.5 × 10^−12^ cm^3^/molecule/s	^18^
7	OH + H + M → H_2_O + M	*α*_*H2O*_ = 1.14 × 10^−10^ cm^3^/mole/s	^19^
8	H + O_2_ + M → HO_2_ + M	*α*_*O2H*_ = 4.6 × 10^−13^ cm^3^/s	^19^
9	OH + OH + M → H_2_O_2_ + M	*α*_*H2O2*_ = 1.78 × 10^−11^ cm^3^/mole/s	^21^
10	OH + HO_2_ → H_2_O + O_2_	*α*_*OH2*_ = 1.1 × 10^−10^ cm^3^/s	^21^
11	OH + H_2_O_2_ → HO_2_ + H_2_O	*α*_*HO2*_ = 1.7 × 10^−12^ cm^3^/s	^21^
12	HO_2_ + HO_2_ → H_2_O_2_ + O_2_	*α*_*H2O2*_ = 1.24 × 10^−12^ cm^3^/molecules/s	^22^
13	H_2_O_2_ + O → HO_2_ + OH	*α*_*HO2*_ = 1.7 × 10^−15^ cm^3^/molecules/s	^21^
14	HO_2_ + H_2_O_2_ → OH + H_2_O + O_2_	1 × 10^−13^ cm^3^/molecules/s	^23^
15	H_2_O_2_ + H → OH + H_2_O	4.2 × 10^−14^ cm^3^/molecules/s	^19^
16	H + N → NH	*α*_*NH*_ = 1.3 × 10^−12^ cm^3^/s	^26^
17	NH + OH → HNO + H	*α*_*HNO*_ = 3.32 × 10^−11^ cm^3^/s	^27^
18	NH + O → OH + N	*α*_*OH*_ = 1.16 × 10^−11^ cm^3^/molecules/s	^28^
19	NH + O_2_ → Products	α_Pro_ = 9.98 × 10^−15^ cm^3^/molecules/s	^26^
20	NH + NH → NH_2_ + N	*α*_*NH2*_ = 4.3 × 10^−13^ cm^3^/s	^29^
21	NH_2_ + OH → NH_2_OH	*α*_*NH2OH*_ = 9.31 × 10^−11^ cm^3^/s	^30^
22	NH_2_ + O → H + HNO	*α*_*H*_ = 7.47 × 10^−11^ cm^3^/molecules/s	
23	O_2_ + NH_2_ → H_2_NOO	*α*_*H2NOO*_ = 1.54 × 10^−15^ cm^3^/molecules/s	
24	H + NH_2_ → NH_3_	*α*_*NH3*_ = 7.7 × 10^−11^ cm^3^/molecules/s	^31^
25	HNO_3_ + NH_2_ → NH_3_ + NO_3_	*α*_*H3N*_ = 3.64 × 10^−13^ cm^3^/molecules/s	^32^
26	NH_3_ + O → OH + NH_2_	*α*_*OHN2*_ = 4.69 × 10^−17^ cm^3^/molecules/s	^27^
27	OH + NH_3_ → H_2_O + NH_2_	*α*_*H2O*_ = 1.6 × 10^−13^ cm^3^/molecules/s	^21^
28	NH_3_ + NO_3_ → HNO_3_ + NH_2_	*α*_*HNO3*_ = 6 × 10^−16^ cm^3^/molecules/s	^33^
29	OH + N → NO + H	*α*_*ON*_ = 4.7 × 10^−11^ cm^3^/molecules/s	^19^
30	N + O → NO	*α*_*NO*_ = 2.39 × 10^−13^ cm^3^/mole/s	^25^
31	N + O_2_ → NO + O	*α*_*NO*_ = 9.22 × 10^−17^ cm^3^/mole/s	^22^
32	NO + H → HNO	*α*_*HNO*_ = 1.56 × 10^−12^ cm^3^/molecules/s	^34^
33	NO + OH → HNO_2_	*α*_*HNO2*_ = 1.78 × 10^−11^ cm^3^/s	^22^
34	HNO_2_ + OH → H_2_O + NO_2_	*α*_*NO2*_ = 5.95 × 10^−12^ cm^3^/s	^21^
35	NO + O → NO_2_	*α*_*NO2*_ = 2.6 × 10^−12^ cm^3^/molecules/s	^35^
36	NO + HO_2_ → OH + NO_2_	*α*_*O2N*_ = 8.85 × 10^−12^ cm^3^/molecules/s	^21^
37	NO_2_ + H → OH + NO	*α*_*HO*_ = 1.47 × 10^−10^ cm^3^/molecules/s	^36^
38	NO_2_ + OH → HNO_3_	*α*_*HNO3*_ = 8.81 × 10^−11^ cm^3^/s	^37^
39	NO_2_ + O → O_2_ + NO	*α*_*O2*_ = 1.03 × 10^−11^ cm^3^/molecules/s	^21^
40	HO_2_ + NO_2_ → HO_2_NO_2_	*α*_*HONO*_ = 4.58 × 10^−12^ cm^3^/molecules/s	^23^
41	HNO_3_ + OH → H_2_O + NO_3_	*α*_*NO3*_ = 1.5 × 10^−13^ cm^3^/s	^35^

We calculated the changes in the chemical reactive species in water according to the O_2_ mole fraction in accordance with experiments.

## Results

### Theoretical Approaches - Chemical species generated in plume of N_2_ plasma jet with O_2_

Various chemical species are generated from the N_2_ plasma jet by changing the O_2_ content in the plasma. The most predominant chemical species in the plasma jet is a metastable state N_2_* [N_2_(A_3_∑_u_^+^)] of excited nitrogen molecules. The rate coefficient *α*_N2*_ is expressed as^16^:

1 $${\alpha }_{N{2}^{\ast }}({T}_{e})=2.25\times {10}^{-10}\sqrt{{T}_{e}}(6.8+2{T}_{e})\,\exp \,(-\frac{6.8}{{T}_{e}})$$

where, *T*_*e*_ is the electron temperature in a unit of eV. Dissociation coefficient of N_2_ by electrons is given by:

2 $${k}_{N}({T}_{e})=4.26\times {10}^{-10}\sqrt{{T}_{e}}(10+2{T}_{e})\,\exp \,(\,-\,10/{T}_{e})$$

Meanwhile, the dissociation coefficient of O_2_by electrons is given by^17^:

3 $${k}_{O}({T}_{e})=4.2\times {10}^{-9}\,\exp \,(\,-\,5.6/{T}_{e})$$

The rate coefficients in Eqs (1–3) increase drastically as the electron temperature *T*_*e*_ increases. The reaction coefficients in Eqs (1–3) are given by *α*_N2*_ = 6.40 × 10^−12^, *k*_*N*_ = 1 × 10^−12^, and *k*_*O*_ = 1.5 × 10^−11^ cm^3^/s for *T*_*e*_ = 1 eV, a typical value of non-thermal plasma.

The excited nitrogen molecules N_2_* return back to the ground state when contact with N_2_, according to N_2_ (A_3_∑_u_^+^) + N_2_ → N_2_ + N_2_ with its reaction coefficient of *α*_*N2*_ = 3 × 10^−18^ cm^3^/molecule/s^18^. The N_2_* returns back to the ground state in contact with O_2_ according to N_2_(A_3_∑_u_^+^) + O_2_ → products with its reaction coefficient of *α*_*O2*_ = 2.5 × 10^−12^ cm^3^/molecule/s^18^. The N_2_* disappears in contact with water molecules^11^ with a dissociation coefficient of *α*_*OH*_ = 5 × 10^−14^ cm^3^/s^18^. In these reactions, gas composition in jet is very important. If the ambient neutral density in the atmospheric pressure at room temperature is *n*_0_, the N_2_ and O_2_ density in the entering gas can be expressed with oxygen mole fraction *ξ*. The O_2_ density *n*_*O2*_ is *ξn*_0_ and the N_2_ density *n*_*N2*_ is (1 − *ξ*)*n*_0_. When the water molecules from the water surface are entering into this mixed gas with its mole fraction of ζ, the H_2_O density *n*_*H2O*_ is ζ*n*_0_. The rate equation of the metastable state density *n*_N2*_ can be calculated from

4 $$\frac{d{n}_{N{2}^{\ast }}}{dt}={\alpha }_{N{2}^{\ast }}(1-\xi )(1-\zeta ){n}_{0}{n}_{p}-[{\alpha }_{N{2}^{\ast }}(1-\xi )+{\alpha }_{O2}\xi +{\alpha }_{OH}\zeta ]{n}_{0}{n}_{N{2}^{\ast }}$$

where *n*_*p*_ is plasma electron density. The saturation time constant *τ*_N2*_ of the metastable state molecules is *τ*_N2*_ = [*α*_*N2*_(1 − *ξ*)*n*_0_ + *α*_*O2*_*ξn*_0_ + *α*_*OH*_*ζn*_0_]^−1^ = [78(1 − ξ) + 6.5 × 10^7^*ξ* + 1.3 × 10^4^ *ζ*]^−1^, which is less than the value of 100 *μs* for *n*_0_ = 2.6 × 10^19^/cm^3^ (the neutral density at ambient temperature in one atmospheric pressure) and *ξ* = 0.001 in a typical N_2_ jet. N_2_ gas at a flow rate of a liter per minute (lpm) is the working gas for the plasma jet, which length is up to a centimeter when the outlet diameter is 1 mm. Since the volume of the plume is about 10^−2^ liter, the fluid element may be in the plasma column for more than 600 *μs*. Hence, metastable state density may be approximated by the steady state value of *n*_N2*_ = *α*_N2*_n_*p*_/[*α*_*N2*_(1 − *ξ*) + *α*_*O2*_*ξ* + *α*_*OH*_*ζ*], which is estimated to be:

5 $${n}_{N{2}^{\ast }}=2.56(1-\xi )(1-\zeta )\eta \times {10}^{12}/(\xi +0.02\zeta +0.0001)$$

particles/cm^3^ for *T*_*e*_ = 1 eV, where the symbol *η* is the normalized plasma density defined by *η* = *n*_*p*_/10^12^. The N_2_* are the beginning of most of the chemical reactions, being proportional to the plasma density. Therefore the overall trends of chemical reactions may not be very sensitive to the plasma density. Remember that the lifetime of N_2_* in a metastable state is longer than 10 *ms*.

### Penetration mechanism of N_2_* in a metastable state into water

We investigate the penetration properties of N_2_* entering into water, when a nitrogen plasma jet injects to a water surface. The N_2_* in the plasma jet may continuously bombard on the water surface, diffuse into water, and generate hydroxyl molecules through a reaction with water molecules^11^ with a dissociation coefficient of *α*_*OH*_ = 5 × 10^−14^ cm^3^/s^18^. Then, the diffusion equation of the N_2_* in steady-state is given by $\nabla {{\Gamma }}_{N{2}^{\ast }}=\frac{d{n}_{N{2}^{\ast }}}{dt}={\alpha }_{OH}{n}_{H2O}{n}_{N{2}^{\ast }}$ ^11^. Here, *n*_*H*20_ is the water density, and Γ_N2*_ is the flux of N_2_* defined by Γ_N2*_ = D∇n_N2*_. The symbol *D* is the diffusion constant of the N_2_*.

The diffusion constant *D* is calculated to be *D* = 6.84 × 10^19^/*n*_*NT*_ in units of cm^2^/s^11^. Introducing ${\kappa }_{{N}_{2}}^{2}=\frac{{\nabla }^{2}{n}_{N{2}^{\ast }}}{{n}_{N{2}^{\ast }}}={\alpha }_{OH}{n}_{H2O}/D$, the diffusion equation for N_2_* in water can be expressed as:

6 $$\frac{{d}^{2}{n}_{N{2}^{\ast }}(z)}{d{z}^{2}}={\kappa }_{{N}_{2}}^{2}{n}_{N{2}^{\ast }}(z)$$

The solution to Eq. (6) is ${n}_{N{2}^{\ast }}(z)={n}_{N0}\,\exp \,(\,-\,{\kappa }_{{N}_{2}}z)$. The density decay length *λ*_*N*_ is calculated to be 15 nm. This means that all N_2_* will be instantaneously converted to hydroxyl and hydrogen atoms.

### Generation of H, OH, HO_2_ and H_2_O_2_ molecules in water

The OH and H molecules that were formed from the disappearance of N_2_* at the water surface make consequence reactions to generate H, OH, HO_2_, and H_2_O_2_. There are many ways to eliminate the H atoms, including OH + H + M → H_2_O + M with its rate coefficient of *α*_*H2O*_ = 4.38 × 10^−30^(*T*_*r*_/*T*)^2^ cm^6^/mole^2^/s = 1.14 × 10^−10^ cm^3^/mole/s in air at T = 300 K^19^, and H + O_2_ + M → HO_2_ + M with its reaction coefficient of: *α*_*O2H*_ = 1.78 × 10^−32^(*T*_*r*_/*T*)^0.8^ cm^6^/mole^2^/s = 4.6 × 10^−13^ cm^3^/s at *T* = 300 K^19^, or *α*_*O2H*_ = 5.71 × 10^−32^(*T*_*r*_/*T*)^1.6^ cm^6^/mole^2^/s = 1.06 × 10^−12^ cm^3^/s at *T* = 300 K^20^. Here M is the neutral particles. There is a considerable difference of *α*_*O2H*_ in the references. However, we use *α*_*O2H*_ in ref.^19^ in the subsequent analysis. Some of the H atoms generated from the N_2_* may diffuse into water, and some of them may diffuse into gas. But most of them may disappear, forming H_2_O and H_2_O_2_ molecules, due to the very high concentration of OH molecules in the water surface. Therefore, in the steady-state case, the H density can be calculated to be:

7 $$\begin{array}{l}{\alpha }_{OH}{n}_{N{2}^{\ast }}{n}_{H2O}-\,{\alpha }_{{\rm{H}}2{\rm{O}}}{n}_{OH}{n}_{H}-{\alpha }_{O2H}{n}_{O2}{n}_{H}=0\\ {n}_{H}\,=\,{\alpha }_{OH}{n}_{N{2}^{\ast }}{n}_{H2O}/(({\alpha }_{O2H}+{\alpha }_{{\rm{H}}2{\rm{O}}}{x}_{OH}){n}_{0})\end{array}$$

where, the symbol *x*_*OH*_ is the ratio of OH density to the ambient air density *n*_0_.

There are many ways of disappearance of hydroxyl molecules. The dominant reactions of OH eliminations are forming H_2_O_2_ with its rate coefficient of *α*_*H2O2*_ = 6.83 × 10^−31^ (*T*_*r*_/*T*)^0.8^ cm^6^/mole^2^/s = 1.78 × 10^−11^ cm^3^/mole/s in air at *T* = 300 K^21^, and combining with HO_2_ with its reaction coefficient of *α*_*OH2*_ = 4.8 × 10^−11^ exp (249/*T*) = 1.1 × 10^−10^ cm^3^/s^21^. Therefore, in the steady-state case, we obtain:

8 $${\alpha }_{OH}{n}_{N{2}^{\ast }}{n}_{H2O}-{\alpha }_{{\rm{H}}2{\rm{O}}2}{n}_{OH}{n}_{OH}-{\alpha }_{H2O}{n}_{OH}{n}_{HO2}=0$$

The major sources of HO_2_ radical composition are H + O_2_ + M → HO_2_ + M with its reaction coefficient of *α*_*O2H*_ = 4.6 × 10^−13^ cm^3^/s^19^, and combination of OH and H_2_O_2_ with its reaction coefficient of *α*_*HO2*_ = 1.7 × 10^−12^ cm^3^/s^21^. The decay of HO_2_ can be represented by OH + HO_2_ → H_2_O + O_2_, with its reaction coefficient of *α*_*OH2*_ = 4.8 × 10^−11^ exp (249/*T*) = 1.1 × 10^−10^ cm^3^/s^21^, which is one of the major destruction mechanism of OH radical. The density of *n*_*HO2*_ radical in steady state can be obtained from:

9 $${\alpha }_{O2H}{n}_{H}\xi {n}_{0}+\,{\alpha }_{{\rm{HO}}2}{n}_{OH}{n}_{H2O2}-{\alpha }_{H2O}{n}_{OH}{n}_{HO2}=0$$

The hydrogen peroxide (H_2_O_2_) molecules are generated from the hydroxyl combination of OH + OH + M → H_2_O_2_ + M, and are eliminated by reaction of OH + H_2_O_2_ → HO_2_ + H_2_O, which are balanced by ${\alpha }_{{\rm{H}}2{\rm{O}}2}{n}_{OH}^{2}={\alpha }_{HO2}{n}_{OH}{n}_{H2O2}$, so the normalized H_2_O_2_ density can be obtained as:

10 $${x}_{H2O2}={n}_{H2O2}/{n}_{0}=({\alpha }_{H2O2}/{\alpha }_{HO2}){x}_{OH}=10{x}_{OH}.$$

And the Eq. (9) is rewritten as to make the normalized HO_2_ density as:

11 $${\alpha }_{O2H}{n}_{H}\xi {n}_{0}+{\alpha }_{{\rm{H}}2{\rm{O}}2}{n}_{OH}^{2}-{\alpha }_{H2O}{n}_{OH}{n}_{HO2}=0$$

12 $${x}_{HO2}=({\alpha }_{H2O2}/{\alpha }_{H2O})({x}_{OH}+{\alpha }_{O2H}{x}_{H}\xi /{\alpha }_{H2O2}{x}_{OH})=0.16({x}_{OH}+0.026{x}_{H}\xi /{x}_{OH}).$$

However, when the hydroxyl density is very small, H_2_O_2_ may be generated from the reaction of HO_2_ + HO_2_ → H_2_O_2_ + O_2_ with its reaction coefficient of *α*_*H2O2*_ = 1.24 × 10^−12^ cm^3^/molecules/s^22^, and disappeared by the reaction of H_2_O_2_ + O → HO_2_ + OH with its reaction of *α*_*O2H*_ = 1.7 × 10^−15^ cm^3^/molecules/s^21^, by HO_2_ + H_2_O_2_ → OH + H_2_O + O_2_ with its reaction coefficient of 1 × 10^−13^ cm^3^/molecules/s^23^, and by the reaction of H_2_O_2_ + H → OH + H_2_O with its reaction coefficient of 4.2 × 10^−14^ cm^3^/molecules/s^19^. Then normalized H_2_O_2_ density can be obtained as $${x}_{H2O2}={n}_{H2O2}/{n}_{0}$$ = $$[({\alpha }_{H2O2}/{\alpha }_{HO2}){x}_{OH}+({\alpha }_{H2O2}/{\alpha }_{HO2}){x}_{HO{2}^{2}}^{2}/{x}_{OH}]$$/$$(1+0.001{x}_{O}/{x}_{OH})$$ = $$[10{x}_{OH}+0.729{x}_{HO{2}^{2}}/{x}_{OH}]/(1+0.001{x}_{O}/{x}_{OH}$$ + $$0.588{x}_{HO2}/{x}_{OH}+0.0247{x}_{H}/{x}_{OH})$$.

Making use of Eqs (5, 7, 8 and 11), we obtain the normalized OH density equation as:

13 $${x}_{OH}^{2}+2b{x}_{OH}-c=0$$

where, the constants b and c are defined by:

14 $$b=4.7\times {10}^{-3}\xi \,;\,c=1.76\times {10}^{-8}\frac{(1-\xi )(1-\zeta )\eta }{\xi +0.02\zeta }$$

The meaningful solution of Eq. (13) is ${x}_{OH}={n}_{OH}/{n}_{0}=\sqrt{{b}^{2}+c}-b$.

Hydrogen atoms are generated through a reaction between the excited nitrogen and water molecules^18^, and N + OH → NO + H^24^, but the reaction between the excited nitrogen and water molecules prevails, due to the high concentration of water. The major elimination of H atoms is H + O_2_ + M → HO_2_ + M with its reaction coefficient of *α*_*O2H*_ = 5.71 × 10^−32^(*T*_*r*_/*T*)^1.6^ cm^6^/mole^2^/s = 1.06 × 10^−12^ cm^3^/s at *T* = 300 K^20^, and forming water by hydrogen atom and hydroxyl^11^ with a reaction coefficient of *α*_*H2O*_ = 1.1 × 10^−10^ cm^3^/s^19^, establishing the hydrogen atom density of ${n}_{H}={\alpha }_{OH}{n}_{N2}{n}_{H2O}/(\,{\alpha }_{O2H}{n}_{O2}+{\alpha }_{H2O}{n}_{OH})$, which can be further simplified to:

15 $${x}_{H}=\frac{{n}_{H}}{{n}_{0}}=5.89\frac{(1-\xi )(1-\zeta )}{(\xi +108{x}_{OH})(\xi \,+0.02\zeta )}\times {10}^{-6}$$

being the normalized H atom density.

**Figure 1:**
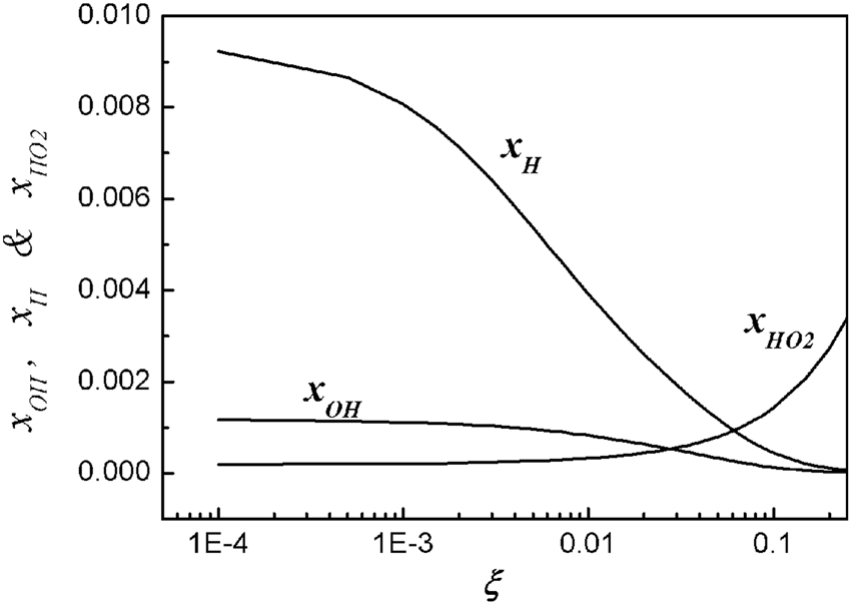
Plots of normalized density of hydroxyl molecule (*x*_*OH*_), hydrogen atom (*x*_*H*_), and hydrogen dioxide (*x*_*HO2*_) versus the oxygen molecular mole fraction *ξ*. The analytical results in Fig. 1 show that as the oxygen mole fraction *ξ* increases from zero to 25%, the hydroxyl molecular density and hydrogen atom density decrease. On the other hand, as the oxygen mole fraction *ξ* increases to *ξ* = 0.25, the hydrogen dioxide density (*x*_*HO2*_) drastically increases.

Figure 1 shows plots of the normalized density of OH, H, and HO_2_ molecules, *x*_*OH*_, *x*_*H*_, *x*_*O*2*H*_, which are normalized by the neutral density at ambient air *n*_0_ = 2.6 × 10^19^/cm^3^. We assume the water density near the water surface is *ζ* = 0.60 (60%), where most of the OH molecules are generated and diffused into water. On the other hand, the H atoms participating in chemical reactions with O and N atoms are borne far away from the water surface, where the water density is about *ζ* = 0.25. The OH molecular density and H atom density decrease as the O_2_ mole fraction *ξ* increases from zero to 0.25. On the other hand, the HO_2_ density increases drastically as the *ξ* increases to 0.25.

### Generation of reactive NH, NH_2_, and NH_3_ molecules in water

For generation of reactive nitrogen species (RNS), the generation of N atom is necessary. Electrons in the N_2_ plasma generate N atoms by impact dissociation of N_2_. The N atoms in the plasma jet may disappear according to the reaction of N + O_2_ → NO + O with its reaction coefficient of *α*_*NO*_ = 9.22 × 10^−17^ cm^3^/mole/s^22^, to the reaction of N + O → NO with its reaction coefficient of *α*_*NO*_ = 2.39 × 10^−13^ cm^3^/mole/s^25^, to the reaction between hydroxyl and nitrogen atom with its reaction coefficient of *α*_*NO*_ = 4.7 × 10^−11^ cm^3^/molecules/s^19^, and to the reaction between hydrogen and nitrogen atoms with a reaction coefficient of *α*_*NH*_ = 1.3 × 10^−12^ cm^3^/s^26^. But most of the OH molecules and H atoms are generated near the water surface. On the other hand, the N atoms are generated at the beginning of plasma jet far away from the water surface. Therefore, the OH molecules and H atoms may not actively participate in the disappearance process of N atoms. Thus the rate equation of the nitrogen atom density *n*_*N*_ is calculated from

$$\frac{d{n}_{N}}{dt}=2{k}_{N}(1-\xi )(1-\zeta ){n}_{0}{n}_{p}-({\alpha }_{O2N}\xi {n}_{0}+{\alpha }_{NO}{n}_{O}){n}_{N}$$

where, the N atom density may not saturate in the plasma column, because of insufficient O_2_ mole fraction. The rate equation of oxygen atom density *n*_*O*_ is given by:

$$\frac{d{n}_{O}}{dt}=2{k}_{O}\xi {n}_{0}{n}_{p}-{\alpha }_{NO}{n}_{O}{n}_{N}$$

where, the O atom density also may not saturate in the plasma jet, continuously growing with time. Assuming the electron temperature of *T*_*e*_ = 1 eV, we find from the above two equations that the N and O atom normalized densities are *x*_*N*_ = 2 × 10^−4^ (1 − *ξ*) (1 − *ς*)*η*/cm^3^ and *x*_*O*_ = 3 × 10^−3^ (1 − *ς*)*ηξ*/cm^3^ at *t* = 100 μs, respectively.

**Figure 2:**
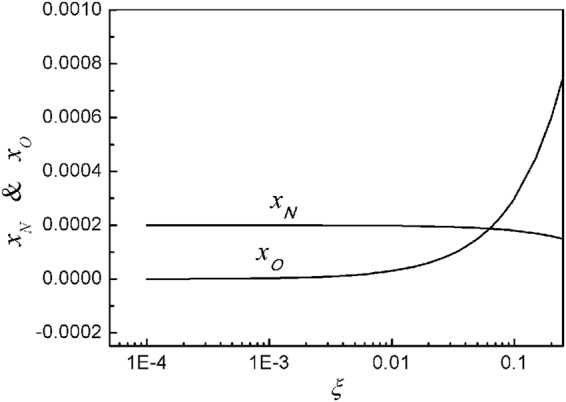
Plots of normalized nitrogen (*x*_*N*_) and oxygen (*x*_*O*_) atoms densities versus oxygen molecular mole fraction of *ξ*. As the oxygen molecular mole fraction of *ξ* increases, the oxygen atom density *x*_*O*_ increases drastically. However, in the entire range of the oxygen mole fraction, the nitrogen atom density is almost constant.

Figure 2 shows plots of the normalized N and O atom densities in terms of the O_2_ mole fraction of *ξ*. The O atom density *x*_*O*_ increases drastically as *ξ* increases. Here, these atom densities are determined at the time *t* = 100 μs, when the plasma jet injects into the water with a limited size of jet length, which is less than 1 cm. The O and N atoms are generated from the beginning very near the jet exit. Hydrogen atoms already start the chemical reactions from this jet exit.

The NH generation process is forming NH by combination of nitrogen and hydrogen atoms, while the major contributions to NH dissociation are the reaction between NH and hydroxyl forming HNO with a reaction coefficient of *α*_*HNO*_ = 3.32 × 10^−11^ cm^3^/s^27^, the reaction between NH and oxygen atom with the reaction coefficient of *α*_*OH*_ = 1.16 × 10^−11^ cm^3^/molecules/s^28^, and the reaction of NH + O_2_ → Products with its reaction coefficient of α_Pro_ = 9.98 × 10^−15^ cm^3^/molecules/s^26^, establishing the steady-state value of ${n}_{NH}=({\alpha }_{NH}/{\alpha }_{HNO})$ $({n}_{H}{n}_{N}/[{n}_{OH}+{\alpha }_{OH}{n}_{O}/{\alpha }_{HNO}+{\alpha }_{pro}{n}_{O2}/{\alpha }_{HNO}])$ rather quickly; therefore, the normalized NH density is expressed as *x*_*NH*_ = 3.9 × 10^−2^ *x*_*H*_ *x*_*N*_/[*x*_*OH*_ + 0.35*x*_*O*_ + 0.0003*ξ*(1 − *ς*)*η*], which is usually less than the N atom density. Combination of two NH molecules form NH_2_^11^ with a reaction coefficient of *α*_*NH2*_ = 4.3 × 10^−13^ cm^3^/s^29^, and NH_2_ molecules are eliminated by reaction between NH_2_ and OH forms NH_2_OH with a reaction coefficient of *α*_*NH2OH*_ = 9.31 × 10^−11^ cm^3^/s^30^, by the reaction of NH_2_ and O forming HNO with its reaction coefficient of *α*_*H*_ = 7.47 × 10^−11^ cm^3^/molecules/s, and by the reaction of O_2_ + NH_2_ → H_2_NOO with its reaction coefficient of *α*_*H2NOO*_ = 1.54 × 10^−15^ cm^3^/molecules/s, leading to a steady-state value of ${n}_{NH2}=({\alpha }_{NH2}/{\alpha }_{NH2OH})$ $({{n}_{NH}}^{2}/[{n}_{OH}+{\alpha }_{H}{n}_{O}/{\alpha }_{NH2OH}+{\alpha }_{H2NOO}{n}_{O2}])$, which can be expressed as *x*_*NH2*_ = *n*_*NH2*_/*n*_0_ = 4.62 × 10^−3^ *x*_*NH*_^2^/[*x*_*OH*_ + 0.8*x*_*O*_ + 1.65 × 10^−5^*ξ*(1 − *ς*)]. The ammonia molecules are generated from the reaction of H + NH_2_ → NH_3_ with its reaction coefficient of *α*_*NH3*_ = 7.7 × 10^−11^ cm^3^/molecules/s^31^, and the reaction between HNO_3_ and NH_2_ forming NH_3_ with its reaction coefficient of *α*_*NH3*_ = 3.64 × 10^−13^ cm^3^/molecules/s^32^. Meanwhile, the ammonia may be eliminated by the reaction of NH_3_ and O with its reaction coefficient of *α*_*OH*_ = 4.69 × 10^−17^ cm^3^/molecules/s^27^, by the reaction between OH and NH_3_^11^ with its reaction coefficient of *α*_*H2O*_ = 1.6 × 10^−13^ cm^3^/molecules/s^21^, and by the reaction of NH_3_ and NO_3_ forming HNO_3_ with its reaction coefficient of *α*_*HNO3*_ = 6 × 10^−16^ cm^3^/molecules/s^33^, leading to a steady-state value of the normalized ammonia density of *x*_*NH3*_ = *n*_*NH3*_/*n*_0_ = 481(*x*_*H*_ + 0.0047*n*_*HNO3*_)*x*_*NH2*_/(*x*_*OH*_ + 0.000293*x*_*O*_), where *x*_*HNO3*_ is the normalized density of nitric acid HNO_3_.

**Figure 3:**
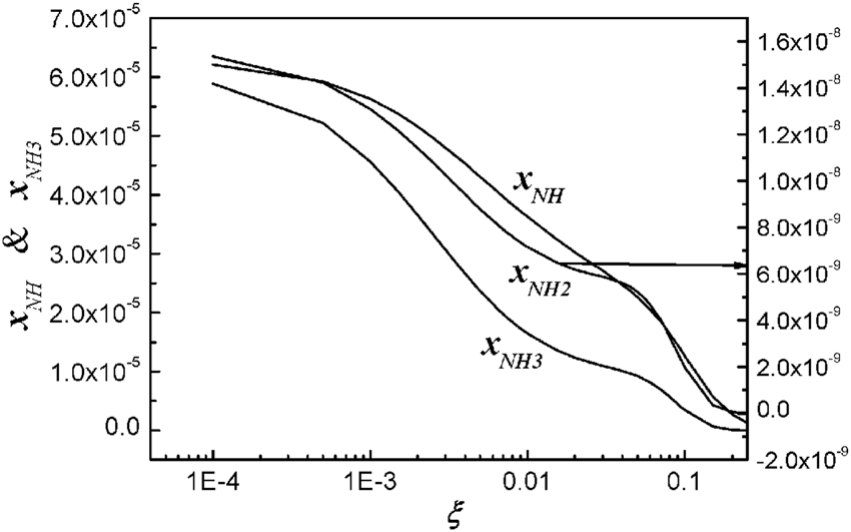
Plots of normalized densities of NH, NH_2_, and NH_3_ molecules versus the oxygen mole fraction of *ξ*. NH, NH_2_, and Ammonia (NH_3_) are well generated in the nitrogen plasma, due to the abundance of hydrogen atoms. However, as the oxygen mole fraction increases, they disappear very quickly. Nevertheless, ammonia generated in the nitrogen plasma jet dissolves into water forming ammonia water, which is a weak alkali.

Figure 3 shows plots of the normalized densities of NH, NH_2_, and NH_3_ molecules in terms of the oxygen mole fraction of *ξ*. Ammonia (NH_3_) is well generated in the N_2_ plasma, due to the abundance of H atoms. However, this compound disappears very quickly, as the O_2_ mole fraction increase. Nevertheless, ammonia generated in the N_2_ plasma jet dissolve into water, forming ammonia water, which is a weak alkali.

#### Generation of reactive NO, HNO_2_, and HNO_3_ molecules in water

We must investigate the nitric oxide behaviors in the environment of high concentration of H atoms in the vicinity of the water surface. Nitric monoxide is formed by the reaction of N + OH → NO + H, by the reaction of N + O → NO, and by the reaction of N + O_2_ → NO + O with its reaction coefficient of *α*_*NO*_ = 9.22 × 10^−17^ cm^3^/mole/s^22^; but its disappearance may be the reaction of NO and H forming HNO with its reaction coefficient of *α*_*HNO*_ = 1.56 × 10^−12^ cm^3^/molecules/s^34^, and the reaction of NO and OH forming HNO_2_ with a reaction coefficient *α*_*HNO2*_ of 1.78 × 10^−11^ cm^3^/s^22^. In this case, the nitrogen monoxide density is given by $${n}_{NO}=({\alpha }_{NO}/{\alpha }_{HNO})({n}_{OH}+{n}_{O}+{\alpha }_{NO2}{n}_{O2}/{\alpha }_{NO}){n}_{N}/({n}_{H}+{\alpha }_{HNO2}{n}_{OH}/{\alpha }_{HNO}+{\alpha }_{NO2}{n}_{O}/{\alpha }_{HNO})$$, which can be expressed as *x*_*NO*_ = 30.1(*x*_*OH*_ + 0.0051*x*_*O*_ + 0.000002*ξ*)*x*_*N*_/(*x*_*H*_ + 11.4*x*_*OH*_ + 1.67*x*_*O*_). The nitrous acid HNO_2_ is destroyed due by the reaction of HNO_2_ and OH with a reaction coefficient of *α*_*HNO2*_ = 5.95 × 10^−12^ cm^3^/s^21^, resulting to the steady-state value of the HNO_2_ density of *n*_*HNO2*_ = 3*n*_*NO*_. The leading reactions of NO_2_ formation are the reaction of NO + O → NO_2_ with its reaction coefficient of *α*_*NO2*_ = 2.6 × 10^−12^ cm^3^/molecules/s^35^, the reaction between HNO_2_ and OH forming NO_2_^11^, and the reaction of NO + HO_2_ forming NO_2_ with its reaction coefficient of *α*_*NO2*_ = 8.85 × 10^−12^ cm^3^/molecules/s^21^. The leading elimination of NO_2_ is the reaction of NO_2_ and H with its reaction coefficient of *α*_*OH*_ = 1.47 × 10^−10^ cm^3^/molecules/s^36^, the reaction of NO_2_ with OH forming HNO_3_ with a reaction coefficient of *α*_*HNO3*_ = 8.81 × 10^−11^ cm^3^/s^37^, the reaction of NO_2_ + O → O_2_ + NO with its reaction coefficient of *α*_*O2*_ = 1.03 × 10^−11^ cm^3^/molecules/s^21^, and the reaction of HO_2_ + NO_2_ → HO_2_NO_2_ with its reaction coefficient of *α*_*HONO*_ = 4.58×10^−12^ cm^3^/molecules/s^23^. Therefore, the steady-state value of NO_2_ density is given by $${n}_{NO2}=({\alpha }_{HNO2}/{\alpha }_{OH})(3{n}_{OH}+3{n}_{OH}+{\alpha }_{NO2}{n}_{O}/{\alpha }_{HNO2}+{\alpha }_{NO2}{n}_{HO2}/{\alpha }_{HNO2})\times \,{n}_{NO}({n}_{H}+{\alpha }_{HNO3}{n}_{OH}/{\alpha }_{OH}+$$$${\alpha }_{O2}{n}_{O}/{\alpha }_{OH}++{\alpha }_{HONO}{n}_{HO2}/{\alpha }_{OH})$$, which is expressed as *x*_*NO2*_ = 0.0405(3*x*_*OH*_ + 0.437*x*_*O*_ + 1.5*x*_*HO2*_)*x*_*NO*_/(*x*_*H*_ + 0.6*x*_*OH*_ + 0.07*x*_*O*_ + 0.0312*x*_*HO2*_). The nitric acid (HNO_3_) can be eliminated by the reaction of HNO_3_ with OH forming NO_3_ with a reaction coefficient of *α*_*NO3*_ = 1.5 × 10^−13^ cm^3^/s^35^, leading to *x*_*HNO3*_ = 587*x*_*NO2*_, and resulting in a very high concentration of nitric acid. We remind the reader that the H atom density *n*_*H*_ is very high for a small mole fraction of oxygen, so that in general, the nitric oxide densities for a small mole fraction of O_2_ are very low. Therefore, the nitric acid density of *n*_*HNO3*_ is low for a small mole fraction of oxygen. On the other hand, the nitric acid density at a high mole fraction of O_2_ is very high for a high value of nitric oxide density.

**Figure 4:**
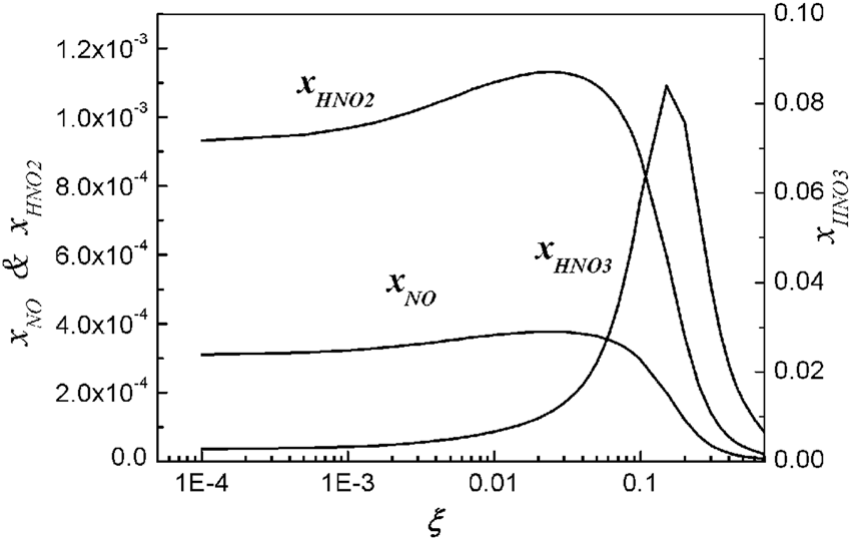
Plots of normalized densities of NO, HNO2, and HNO3 versus the oxygen mole fraction of *ξ*. At a small mole fraction of oxygen, the nitric acid density (*x*_*HNO3*_) is low; but as the oxygen mole fraction increases, its intensity increases to a peak value of around *ξ* ≈ 0.2, and then deceases. Meanwhile, in the range of *ξ* ≤ 0.25, the densities of HNO_2_ and NO are at a moderate level. The entire range of the oxygen mole fraction shows the existence of a moderate level of nitrogen monoxide (NO).

Figure 4 shows estimations of the nitric acid compounds in terms of the O_2_ mole fraction. The nitric acid density (*x*_*HNO3*_) is low at a small mole fraction of oxygen, but its intensity increases to a peak value of around *ξ* ≈ 0.2, and then deceases, as the *ξ* increases. Meanwhile, the densities of HNO_2_ and NO are at moderate levels in the range of *ξ* ≤ 0.25. Note that HNO_3_ is a strong acid; meanwhile, HNO_2_ is a weak acid. We remind the reader that a moderate level of nitrogen monoxide (NO) exists in the entire range of the O_2_ mole fraction.

### pH change in water activated by nitrogen plasma jet with oxygen

The ammonia can be dissolved into water forming NH_4_OH, which turns the water alkali, increasing the *pH* value of the water. Meanwhile, the nitric acid HNO_3_ also dissolves into water, making the water acidic with reducing *pH* value. Assuming the initial deionized water with its *pH* value of *γ*, the initial mole fractions of H^+^ and OH^−^ ions are given by [H^+^] = 10^−γ^ and [OH^−^] = 10^γ−14^. Note that NH_4_OH is a weak alkali, so that its electrolysis in water is partial. On the other hand, HNO_3_ is a strong acid, so that its electrolysis in water is full. In this context, it is very difficult to analytically determine the *pH* value of water mixed with NH_4_OH and HNO_3_. However, we observed from Figs 3 and 4 that the ammonia (*x*_*NH3*_) dominates over the nitric acid (*x*_*HNO3*_) at a very small value of the O_2_ mole fraction of *ξ* ≤ 10^−4^, being alkali. On the other hand, the intensity of nitric acid is strong for the O_2_ mole fraction of *ξ* ≥ 0.01, turning the plasma activated water to acidic.

## Experimental Approaches

We compare the theoretical results with the experimentally measured data.

**Figure 5:**
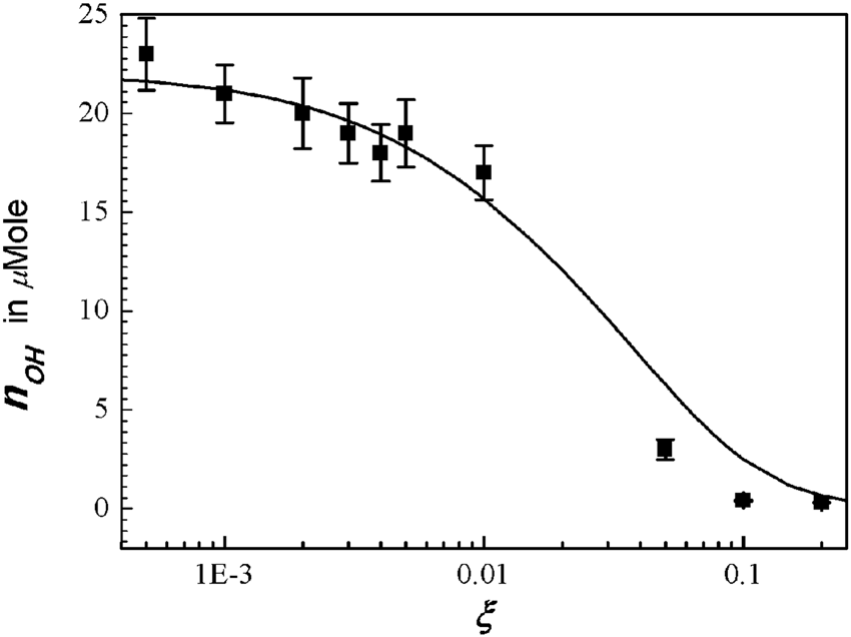
Plot of hydroxyl density in deionized water activated by the nitrogen plasma jet versus the oxygen mole fraction of *ξ*. Dots in the figure are the experimental data from the averaged value of three times of measurements. The hydroxyl molecules are mostly generated at water surface, and some of them can penetrate into water. The theoretical result (curve) obtained from ${x}_{OH}={n}_{OH}/{n}_{0}=\sqrt{{b}^{2}+c-b}$ is least-squared-fitted to the experimental data, where the symbols *b* and *c* are expressed in Eq. (14) in terms of the oxygen mole fraction *ξ*. The typical error bar is shown in the data at the oxygen mole fraction *ξ* = 0.003, where the size of the error bar due to the experimental process is about 8% of its measurement value. At a small mole fraction of oxygen, the hydroxyl density is strong; but as the oxygen mole fraction increase to 0.2, corresponding to the oxygen mole fraction of air, it quickly disappears.

Figure 5 is a plot of the OH density in deionized water activated by the N_2_ plasma jet. Most of the hydroxyl molecules are generated at water surface, and some of them can penetrate into water. We do not know what fraction of hydroxyl molecules are injected into the water. Therefore, the theoretical result obtained from ${x}_{OH}={n}_{OH}/{n}_{0}=\sqrt{{b}^{2}+c}-b$ is least-squared-fitted to the experimental data, where the symbols *b* and *c* are expressed in Eq. (14) in terms of the O_2_ mole fraction *ξ*. Typical error bar is shown in the data at *ξ* = 0.003, where the size of the error bar due to the experimental process is about 8% of its measurement value. Every experimental datum was determined by three times of measurements. Figure 5 clearly shows that the hydroxyl density is strong at a small *ξ*, but it quickly disappears as *ξ* increases to 0.2, corresponding to the O_2_ mole fraction of air.

**Figure 6:**
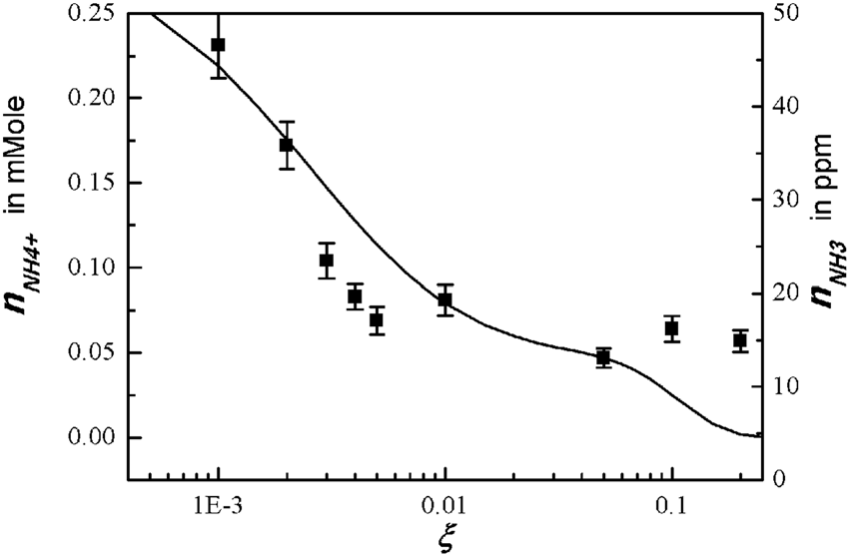
Plot of experimental data of NH_4_^+^ ion concentration in water versus oxygen mole fraction of *ξ*. The ammonia of NH_3_ may dissolve into water forming ammonia water, where NH_4_^+^ ions may be generated. The theoretical result (curve) of ammonia obtained from *x*_*NH3*_ = *n*_*NH3*_/*n*_0_ = 481(*x*_*H*_ + 0.0047*n*_*HNO3*_)*x*_*NH2*_/(*x*_*OH*_ + 0.000293*x*_*O*_) or from Fig. 3 is least-squared-fitted to the experimental data of NH_4_^+^ concentration. The typical error bar is shown in the data at the oxygen mole fraction *ξ* = 0.01, where the size of the error bar due to experimental process is about 11% of its measurement value. The experimental data follows the trend of the theoretical results. As the oxygen mole fraction increases to *ξ* = 0.2, the ammonia concentration decreases.

Figure 6 is the experimental data of the NH_4_^+^ ion concentration in water in terms of the O_2_ mole fraction *ξ*. Based on a previous report^38^, we assume that the ammonia of NH_3_ may dissolve into water forming ammonia water, where NH_4_^+^ ions may be generated. The concentration of NH_4_^+^ ions plays an important role in determination of *pH* value in water. Therefore, the theoretical result (curve) of ammonia obtained from *x*_*NH3*_ = *n*_*NH3*_/*n*_0_ = 481(*x*_*H*_ + 0.0047*n*_*HNO3*_)*x*_*NH2*_/(*x*_*OH*_ + 0.000293*x*_*O*_) or from Fig. 3 will be least-squared-fitted to the experimental data of NH_4_^+^ concentration. The vertical axis in the right represents the ammonia concentration in the plasma jet in ppm unit. The typical error bar is shown in the data at *ξ* = 0.01, where the size of the error bar due to the experimental process is about 11% of its measurement value. The experimental data follows the trend of the theoretical results. The ammonia concentration decreases as *ξ* increases to 0.2.

**Figure 7:**
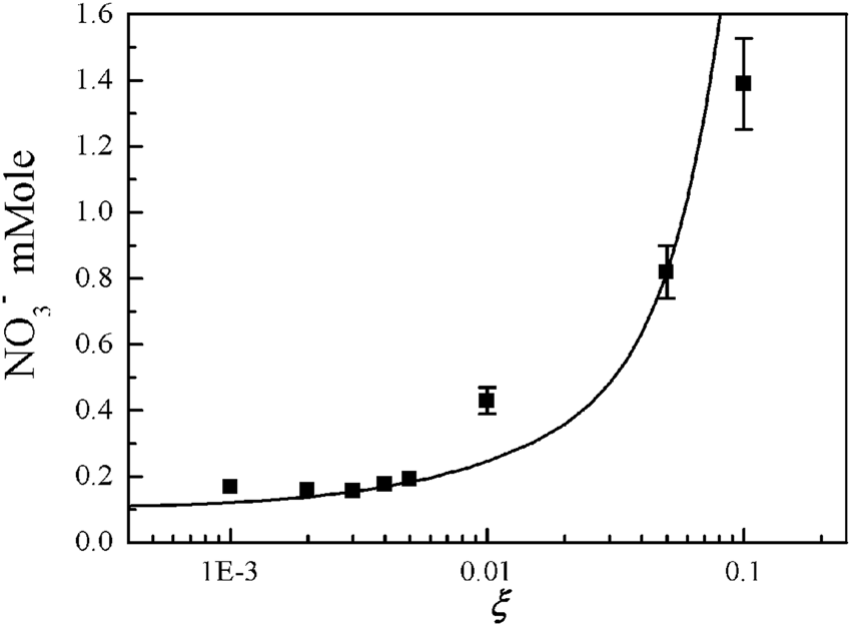
Plots of the experimental data of NO_3_^−^ ion concentration in water versus oxygen mole fraction of *ξ*. The theoretical results in Fig. 4 are least-squared-fitted to the experimental data. The typical error bar is shown in the data at the oxygen mole fraction *ξ* = 0.05, where the size of the error bar due to the experimental process is about 10% of its measurement value. As the oxygen mole fraction of *ξ* increases to *ξ* = 0.2, the experimental data and theoretical result (curve) indicate the increase of nitric acid. However, the theoretical curve in Fig. 4 indicates that as the oxygen mole fraction increases beyond *ξ* = 0.2, the nitric acid (HNO_3_) deceases drastically.

Figure 7 is the experimental data of the NO_3_^−^ ion concentration in water, in terms of the O_2_ mole fraction of *ξ*. The theoretical results (curve) obtained from Fig. 4 in terms of HNO_3_ are also plotted in this figure, reasonably assuming that the nitric acid of HNO_3_ is dissolving into water. The theoretical results are least-squared-fitted to the experimental data. The typical error bar is shown in the data at *ξ* = 0.05, where the size of the error bar due to the experimental process is about 10% of its measurement value. It is obvious from Fig. 7 that the experimental data and theoretical result indicate the increase of nitric acid as *ξ* increases to 0.2. However, the theoretical curve in Fig. 4 indicates that as the *ξ* increases beyond 0.2, the nitric acid (HNO_3_) deceases drastically.

**Figure 8:**
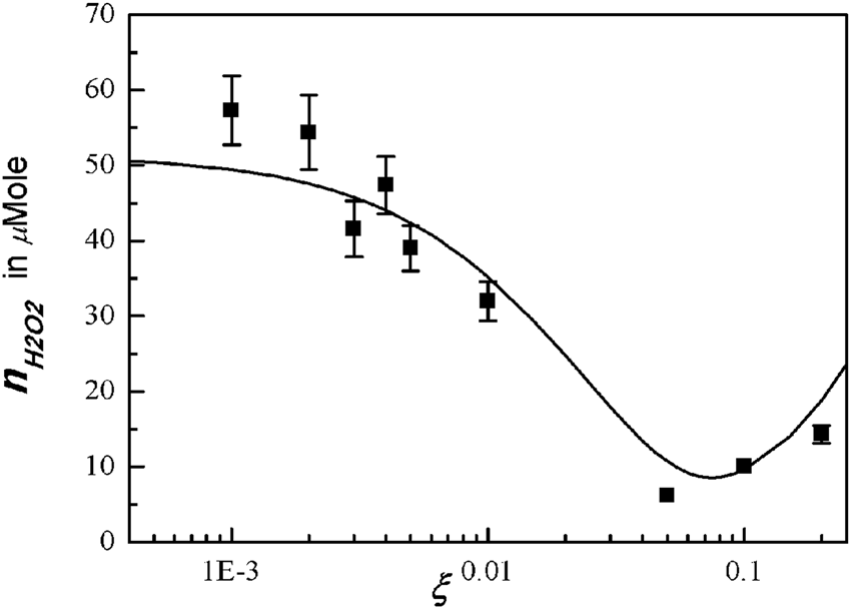
Plot of experimental data (dots) of the hydrogen peroxide versus the oxygen mole fraction *ξ*. At a small value of oxygen mole fraction, the intensity of the hydrogen peroxide is very high, where a relatively high intensity of hydroxyl generates H_2_O_2_, as expected from Fig. 1. But as the oxygen mole fraction increases, its intensity decreases, like the hydroxyl density. However, as the oxygen mole fraction increases to a large value, the hydrogen peroxide density increases again, due to the strong surge of hydrogen dioxide molecules shown in Fig. 1. Therefore, as the oxygen mole fraction increases from a small value to a large value, the hydrogen peroxide density deceases, reaches its minimum value at *ξ* = 0.05, and then increases again.

One of the most important reactive chemicals in water is the H_2_O_2_ generated from N_2_ plasma jet. Figure 8 shows a plot of the measurement data (dots) of the H_2_O_2_ in water activated by a N_2_ plasma jet with changing O_2_ mole fraction of *ξ*. The theoretical curve obtained from *x*_*H2O2*_ = *n*_*H2O2*_/*n*_0 = _[10*x*_*OH*_ + 0.729*x*_*HO2*_^2^/*x*_OH_]/(1 + 0.001*x*_*O*_/*x*_*OH*_ + 0.588*x*_*HO2*_/*x*_*OH*_ + 0.0247*x*_*H*_/*x*_*OH*_) is least-squared-fitted to the experimental data. The typical error bar is shown in the data at *ξ* = 0.005, where the size of the error bar due to the experimental process is about 9% of its measurement value. The intensity of the H_2_O_2_ is very high at a small value of *ξ*, where a relatively high intensity of hydroxyl generates H_2_O_2_, as expected from Fig. 1. But similar to the hydroxyl density, its intensity decreases as *ξ* increases. However, the H_2_O_2_ density increases again as *ξ* increases to a large value. The rebounding increase of H_2_O_2_ at a large value of *ξ* is caused by a strong surge of HO_2_ molecules shown in Fig. 1, which generate H_2_O_2_ molecules. Therefore, the density of H_2_O_2_ deceases, reaches its minimum value at *ξ* = 0.05, and then increases again, as the *ξ* increases from a small value to a large value. The experimental data follow the theoretical trend, but there are some deviations from the theoretical curve. Further study is needed to resolve this difference in future.

**Figure 9:**
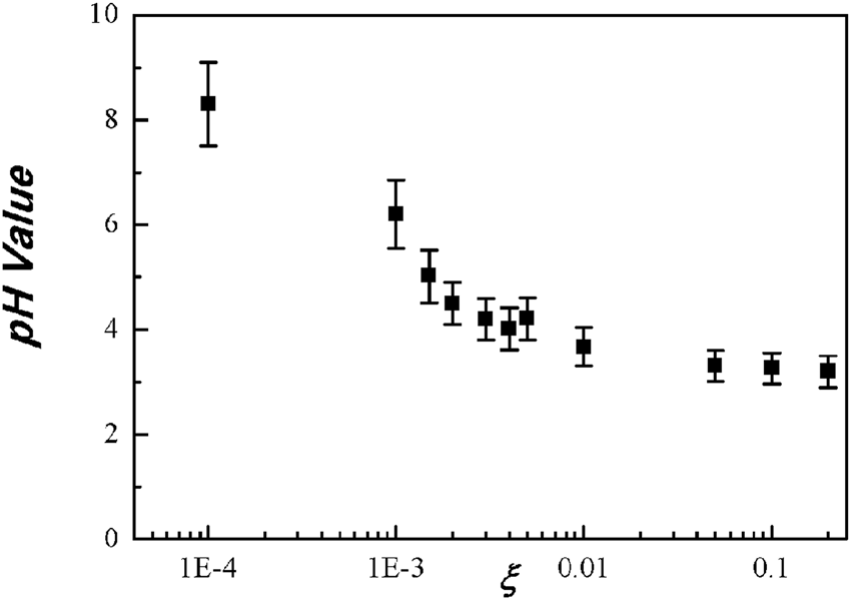
Plots of experimental data of the *pH* values of water activated by the nitrogen plasma versus the oxygen mole fraction of *ξ*. The typical error bar in this experiment is shown in the data at the oxygen mole fraction *ξ* = 0.01, where the size of the error bar due to measurement process is about 10% of its measurement value. As expected in the analytical study, at a very small value of the oxygen mole fraction, the plasma activated water is alkali, and as the oxygen mole fraction increases to a large value, it becomes acidic The *pH* of the plasma activated water at *ξ* = 0.2 corresponding to air is about 3.

Finally, we like to investigate the *pH* of water activated by the nitrogen plasma by changing the oxygen mole fraction of *ξ*. Figure 9 is the experimental data of the *pH* values of water activated by the nitrogen plasma. The *pH* value was measured in terms of the oxygen mole fraction. The typical error bar is shown in the data at the oxygen mole fraction *ξ* = 0.01, where the size of the error bar due to measurement process is about 10% of its measurement value. As expected in the analytical study, the plasma activated water is alkali at a very small value of the oxygen mole fraction, due to the high density of ammonia, and then it becomes acidic as the oxygen mole fraction increases to a large value. The *pH* of the plasma activated water at *ξ* = 0.2 corresponding to air is about 3.

## Discussion

The purpose of this study is the investigation of the influence of O_2_ on the generation of reactive chemical species from a N_2_ plasma jet near a water surface. The most abundant reactive species in a N_2_ plasma jet is the excited nitrogen molecules in the metastable state of N_2_*, which in turn dissociate water molecules, generating hydroxyl (OH) molecules and hydrogen atoms near the water surface. A presence of oxygen molecules may obstruct the dissociation mechanism of water molecules by the excited nitrogen molecules. In this regard, we theoretically and experimentally investigate the reactive chemical species in nitrogen plasma by changing the O_2_ mole fraction of *ξ*.

Various chemical compounds are fabricated from N_2_* and water molecules in plasma jet with varying O_2_ content. Detailed theoretical investigation of these chemical compounds is carried out in terms of different O_2_ content. Hydroxyl molecules and hydrogen atoms are well fabricated near the water surface by a nitrogen plasma jet without oxygen. But Fig. 1 shows that the densities of those species decrease as *ξ* increases. On the other hand, the density of the hydrogen dioxide increases drastically as *ξ* increases, turning the hydroxyl molecules into water. Due to the high density of hydrogen atoms, the ammonia intensity is relatively high at a small value of *ξ*. But Fig. 3 shows that the ammonia density (*x*_*NH3*_) decreases drastically as *ξ* increases. Nitrogen monoxide (NO) is one of the important molecules related to a signaling material in cells. Figure 4 shows that the density of nitrogen monoxide is at a moderate level over the entire range ξ ≤ 0.2. Nitric acid (HNO_3_) starts from a negligibly small value, increases to a peak value at *ξ* ≈ 0.2, and then decreases, as *ξ* increases to a large value.

Electrons in plasma jet may have active roles in electron-impact ionization, excitation, dissociations, etc. There are hundreds chemical reactions of N, H, O elements in “plasma and humid air” or “plasma at water boundary” with electrons involved. Some of these reactions may play important roles. Investigating all of these reactions may be beyond the scope of this article. We therefore consider the most important reactions associated with negative affinity of oxygen. As a first example, water molecules made of oxygen may undergo a dissociative attachment by the reaction of e + H_2_O → H^−^ + OH with the dissociative-attachment coefficient of *α*_*da*_. This reaction is important because of hydroxyl production. However, the excitation cross section of nitrogen molecules is one order in magnitude larger than the dissociative-attachment cross section of water, thereby estimating to be *α*_N2*_ = 6.4 × 10^−12^ cm^3^/s and *α*_*da*_ = 9 × 10^−13^ cm^3^/s at *T*_*e*_ = 1 eV^17,39^. The dominant gas in the plasma jet is nitrogen molecules so that the density of the metastable state N_2_* is 2.6 × 10^16^/cm^3^ due to a long lifetime^11^, dissociating water molecules by reaction of N_2_(A_3_∑_u_^+^) and H_2_O with a dissociation coefficient^19^ of *α*_*OH*_ = 5 × 10^−14^ cm^3^/s. On the other hand, the electron density may be considerably less than 10^12^/cm^3^ as approaching water surface due to various reasons including spreading, recombination, electron attachment, diffusions, etc. The product of (2.6 × 10^16^/cm^3^) (5 × 10^−14^ cm^3^/s) is 1.3 × 10^3^/s, whereas the product of (10^12^/cm^3^) (9 × 10^−13^ cm^3^/s) is 9 × 10^−1^/s. Therefore, the dissociative attachment of water molecules by electrons may be negligible in comparison with water dissociation by N_2_(A_3_Σ_u_^+^). The other important reaction of the electron affinity is the dissociative attachment of oxygen molecules by e + O_2_ → O^−^ + O with dissociative attachment coefficient of *k*_*da*_, which is less than one fifth of *k*_*O*_ in Eq. (3) at *T*_*e*_ = 1 eV^40^.

Experimental measurements of the reactive chemical species in the plasma activated water were also carried out by comparison with the theoretical results. This identified that hydroxyl molecules are mostly generated at water surface, and some of them can penetrate into water. The hydroxyl molecular density reaches its maximum without oxygen, and decreases to zero, as *ξ* increases to 0.25, showing that the theoretical prediction agrees reasonably well with the experimental data. The theoretical results and experimental data also indicate that the density of the ammonia of NH_3_ also deceases as *ξ* increases to 0.25. On the other hand, theory and experiment show that the density of the NO_3_ increases drastically as *ξ* increases to 0.25. The density of hydrogen peroxide in plasma activated water was measured. The hydrogen peroxide density deceases, reaches its minimum value at *ξ* = 0.05, and then increases again, as *ξ* increases from a small value to a large value. Although the experimental data of the hydrogen peroxide follow the theoretical trend, there are considerable deviations from the theoretical curve. Further study is therefore recommended in future to resolve this difference. The *pH* value of the water activated by the N_2_ plasma jet is experimentally measured. The *pH* value of the plasma activated water, which is slightly alkali without oxygen, decreases to three as *ξ* increases to 0.25, which can be expected by the nitric acid in Fig. 4, and was also confirmed by experiments. The *ξ* of ambient air is about 0.2, so that the *pH* value of water activated by the air plasma is acidic, with *pH* ≈ 3.

## Method

### Generation of the nitrogen plasma jet

A nonthermal plasma device is operated at atmospheric pressure inside a vacuum chamber filled with N_2_ gas^41^. The plasma jet system is powered by a 60 Hz AC power supply using a neon transformer (PNP-1000, Daekwang Electric Co.). The inner electrode is a stainless-steel cylinder with an inner and outer diameters of 1.2 mm and 1.4 mm respectively, which is covered by a quartz tube with an outer diameter of 3.2 mm. The outer electrode is fabricated from stainless steel, and is centrally perforated with a hole of 1 mm, through which the plasma jet is ejected to the water surface in a dish surrounded by N_2_ gas. The microdischarges in the porous alumina between inner and outer electrodes evolved into a plasma jet as the applied power increased. Significant changes in the discharge voltage and current waveforms were observed during the process of the evolution to the plasma jet. The current pulses were of short durations of 30–100 ns in the close-up image. They had repetition rates of 10–400 kHz and amplitudes reaching a few amperes and the discharge voltage of a few kV. This indicates that even at a frequency as low as 60 Hz, the plasma that evolves from a large amount of microdischarge inside a porous dielectric can have characteristics that are similar to those generated at several hundreds of kilohertz. The ratios of nitrogen and oxygen gases are controlled by a mass flow controller (GMC 1200, Atovac), and the total gas flow rate through the device is 1 liter per minute (lpm). In order to avoid the influence of O_2_ in the surrounding air, the experiment is carried out in a glove box, where the O_2_ concentration is kept at less than 1%.

One of the most important issues in plasma jet is plasma properties represented by the electron temperature *T*_*e*_ and density *n*_*p*_, which can vary very sensitively by oxygen mole fraction*ξ*. The electron temperature increases as the oxygen mole fraction increases, whereas the electron density decreases instead^42,43^. The electron temperature *T*_*e*_ and density *n*_*p*_ may also be functions of measurement time and space in the plasma jet. We measured the electron temperature and density very close to the jet injection point near the electrodes. The electron temperature *T*_*e*_ is measured to be approximately 0.5 eV for *ξ* = 0 and 1.5 eV for *ξ* = 0.2, consistent to data in refs^42,43^, whose experimental setups are very similar configuration to the present experiment. We therefore assume the electron temperature to be *T*_*e*_ = 1 eV in analytical calculation in our theoretical model. On the other hands, the plasma electron density varies in wide range according to the measurement point in the plasma jet, although the plasma density decreases drastically as the oxygen mole fraction increases, as expected. The electron density for *ξ* = 0.2 (air) is significantly less than that for *ξ* = 0 (nitrogen only) and is localized near electrodes even for higher discharge voltage due to electron attachment of oxygen molecules. The theoretical results in the analytical calculation based on the assumption of the electron density to be *n*_*p*_ = 10^12^/cm^3^ are least-squared-fitted to the experimental data in qualitative comparison, observing agreement between theoretical trend and experimental data. A study of plasma evolution in time and space for a given oxygen mole fraction is beyond the scope of present research and will be left for future work for customized investigation of individual experimental configurations.

### Measurement of various chemical species and *pH* values in water

The deionized (DI) water is deoxygenized by N_2_ purging before experiments, and 1 ml DI water is placed 5 mm below the electrode. DI water is treated with nonthermal plasma for 3 minutes, and the concentrations of OH∙, H_2_O_2_, NO_2_^−^, NO_3_^−^, and NH_4_^+^, and the pH are measured. For OH∙ radical measurement, terephthalic acid (TA) that specifically reacts with OH∙ to become fluorescent hydroxyl-terephthalic (HTA) is used. Both TA (185361, Sigma-Aldrich Co) and HTA (752525, Aldrich) are solved in 35 mM NaOH solution to make 10 mM solutions, and the diluted HTA solution in TA solution is used as a standard to quantify OH∙ impinging on the TA solutions. The fluorescence intensity is measured using a spectrophotometer with a filter set of 340/420 nm (ex/em). For H_2_O_2_ measurement, Amplex UltraRed reagent (A36006, Invitrogen) is used following the manufacturer’s protocol. NO_3_^−^ and NH_4_^+^ ion concentrations are detected by ion chromatography method. The pH value is measured using a pH meter (pH Spear, Eutech Instruments). All liquid samples are treated by the APNP under the same conditions.
